# Inhibition of Colorectal Cancer Cell Proliferation by Regulating Platelet-Derived Growth Factor B Signaling with a DNA Aptamer

**DOI:** 10.31557/APJCP.2019.20.2.487

**Published:** 2019

**Authors:** Suvaraporn Sae-Lim, Boonchoy Soontornworajit, Pichayanoot Pichayanoot

**Affiliations:** 1 *Department of Preclinical Science, Faculty of Medicine,*; 2 *Division of Chemistry, Faculty of Science and Technology, Thammasat University, Pathumthani, Thailand. *

**Keywords:** Colorectal cancer, DNA aptamer, ERK1/2, platelet, derived growth factor B

## Abstract

**Background:**

Overexpression of platelet-derived growth factor-BB (PDGF-BB) is associated with colorectal carcinogenesis. PDGF-BB plays a role in the autocrine growth stimulation of cancer cells. Aptamers are short single-stranded oligonucleotides that can bind to cellular targets with high affinity and specificity and offer the advantage of non-immunogenicity, non-toxicity and high stability. Thus, they receive interest as potential therapeutic agents.

**Methods:**

The endogenous level of PDGF-BB in Caco-2 and SW480, colorectal cancer (CRC) cells, was evaluated using ELISA. The effect of the PDGF-BB aptamer on cell proliferation was investigated in two CRC cell lines and CCD841 CoN, normal colon cells. The effective molar ratio between PDGF-BB and PDGF-BB aptamer was further explored. Cell viability in all experiments was analyzed using MTS assay. Western blotting was performed to examine the alteration of relevant signaling pathways.

**Results:**

Caco-2 and SW480 cells endogenously synthesized and secreted PDGF-BB to stimulate their growth. Cells treated with the PDGF-BB aptamer proliferated at a slower rate, but CCD841 CoN did not. Pre-incubation of PDGF-BB with the corresponding aptamer at the molar ratio 1:1 could significantly silence its proliferative effect on CRC cells. Western blot analysis revealed that the phosphorylation level of ERK1/2, a key component in PDGF downstream signaling pathway, was down-regulated by the aptamer, indicating the underlying mechanism of inhibition of CRC cell proliferation.

**Conclusions:**

This study demonstrated that using a DNA aptamer to interfere with the binding of PDGF-BB to its receptor suppressed CRC cell proliferation in part via down-regulation of the Ras/Raf/MEK/ERK signaling pathway. It raised the possibility that the PDGF-BB-specific aptamer could be a promising therapeutic agent for CRC targeted therapy.

## Introduction

Colorectal cancer (CRC) is the third most common cancer in men and the second most common in women worldwide (Ferlay et al., 2013). Patients with CRC present are found to be at a metastatic phase at the time of diagnosis, or they will develop metastases later; consequently, the mortality rate reaches approximately 45% (Schmoll et al., 2012). The practical treatment for CRC is a combination of surgery, radiation therapy, chemotherapy and biological therapy which can prolong the overall survival rate; however, the clinical challenges are the poor prognosis of advanced and recurrent CRC (Gollins, 2010). The treatment also has limitations, for example, drug resistance and severe side effects. Those limitations result in suffering from infection, fatigue, and diarrhea (Leslie, 2016).To improve overall treatment efficacy, new approaches and novel drug targets should be explored and focused on several molecular pathways to understand the pathophysiological mechanisms leading to the development of CRC.

In this study, platelet-derived growth factors (PDGFs) are the targets of interest because they play important roles in cancer cell growth, angiogenesis and metastasis. PDGFs are growth factors derived from platelets, endothelial and cancer cells. They can promote proliferation and angiogenesis through the PDGF signaling pathway in a variety of cancer types including breast (Pinto et al., 2014), bone (McGary et al., 2002), prostate (Sintich et al., 1999), and colorectal cancers (Yuge et al., 2015). Overexpression of PDGFs is a risk factor for developing CRC and increases the severity of the disease (Ross et al., 1993). There are five isoforms of PDGFs (AA, AB, BB, CC, and DD). In CRC, PDGF-BB-transfected cells showed a significant difference in growth rates *in vivo* (McCarty et al., 2007). CRC patients with a high level of PDGF-BB expression have lower survival rates and more vascular invasion than patients with a low expression (Nakamura et al., 2008). Key pathways immediately downstream of the PDGF signaling include Ras/Raf/MEK/ERK and PI3K/PTEN/Akt/mTOR pathways. Both pathways eventually converge on the nucleus to drive proliferation, survival, angiogenesis, and metastasis and invasion of CRC cells (Cohen et al., 2005; Fang and Richardson, 2005; Steelman et al., 2011).

There are different types of molecules that can bind to PDGF isoforms such as antibodies, aptamers, and soluble extracellular parts of the receptors. These molecules can prevent the PDGFs from binding to their receptors, subsequently inhibiting the downstream signaling pathway (Green et al., 1996; Hawthorne et al., 2008). The aptamers are molecules of interest because of their recognition ability to bind related targets with high specificity and affinity (Ulrich and Wrenger, 2013). Conventionally, aptamers are short single-stranded DNAs or RNAs identified by the process named as a systematic evolution of ligands by exponential enrichment (SELEX) (Ellington and Szostak, 1990). They also demonstrate a low immunogenicity and toxicity (Ulrich and Wrenger, 2013). The aptamers are promising therapeutic molecules for diseases that the extracellular blockade of protein–protein interactions is required. The therapeutic mechanism commonly relies on the inhibition of ligand-receptor interaction by aptamer as antagonist. As mentioned above, regulation of PDGF signaling is a potential target for cancer treatment (Claesson-Welsh, 2012). Binding interaction between PDGFs and/or PDGFR and its corresponding aptamer might address this regulation strategy. An aptamer that binds with high affinity to PDGF-BB, a critical isoform in colorectal carcinogenesis, has been screened for use as antagonist since 1996 (Green et al., 1996). Most of the application for this aptamer focused on quantitative analysis (Xie and Walton, 2010), drug delivery system (Battig et al., 2012) and controlled-release materials (Soontornworajit et al., 2010) for its growth factor target. Recently, an anti-PDGF aptamer, called E10030 has been examined in a phase IIb clinical trial for the treatment of neovascular age-related macular degeneration (nAMD) (Jaffe et al., 2017). However, there are no reports demonstrating roles of the aptamer as PDGF antagonist in CRC treatment. 

In the present work, we aimed to investigate the effects of the PDGF-BB-specific aptamer on CRC cell proliferation and the alteration of downstream signaling pathways. We found that PDGF-BB was endogenously produced and secreted by CRC cell lines: Caco-2 and SW480. Treatment of exogenous PDGF-BB was able to stimulate CRC cell growth, while pre-incubating the growth factor with the aptamer abrogated the growth stimulation. In addition, the efficient interruption of PDGF-BB signal transduction by aptamers was demonstrated by the reduction of the ERK1/2 signaling pathway. Our study results provided a valuable preliminary step in developing the PDGF aptamer as a drug candidate for CRC therapy.

## Materials and Methods

*Reagents*

Recombinant human platelet-derived growth factor subunit B (PDGF-BB, MW = 24.6 kDa) was purchased from R and D Systems (USA). It was dissolved in 4 mM HCl as a concentration of 100 µg/mL stock solution and stored at -20^o^C. The PDGF-BB aptamer was purchased from Integrated DNA Technologies (USA). The sequences are shown as follows: ATC CGC CTG ATT AGC GAT ACT CCA CAG GCT ACG GCA CGT AGA GCA TCA CCA TGA TCC TGT GAC TTG AGC AAA ATC ACC TGC AGG GG (Green et al., 1996). It was dissolved in sterile distilled water as a concentration of 100 µM stock solution and stored at -20^o^C. The following rabbit monoclonal antibodies were purchased from Cell Signaling Technology (USA): Akt (#4691S), phospho-Akt (#4060S), phospho-ERK1/2 (#4370S), ERK (#4695S), and alpha tubulin (#2144S) and diluted 1: 1,000 in diluent solution. Odyssey blocking buffer and goat anti-rabbit IRDye 800CW secondary antibody (#926-32211) were purchased from Li-COR (USA). The working dilution of secondary antibody was 1: 10,000 in diluent solution. Akt Control Cell extracts (#9273) were received from Cell Signaling Technology (USA).

*Cell* *culture*

Two types of human colorectal adenocarcinoma cell lines (SW480 and Caco-2) and the normal colorectal cell line (CCD841 CoN) were purchased from American Type Culture Collection (ATCC, USA). Cells were cultured in Dulbecco’s Modified Eagle’s media (Gibco, USA) supplemented with 10% fetal bovine serum (Gibco, USA) and 1% Penicillin-streptomycin (Gibco, USA) at 37^o^C and 5% CO_2_ humidified atmosphere. Cells were passaged using 0.05% trypsin/EDTA.

*Quantitation of endogenous PDGF-BB level in CRC cells*

SW480 and Caco-2 cells were seeded into 6-well plates at 3 x 10^5^ cells/well in culture media supplemented with 10% FBS. First, the media were collected and centrifuged at 3,000 rpm for 5 minutes at 4^o^C. The supernatants were collected on ice for further analysis. Second, cells were harvested and washed with cold PBS three times. The cell pellets were added 120 µl of RIPA buffer (Amresco, USA) with protease inhibitors (Amresco, USA), homogenized using a sonicator, and centrifuged at 15,000 rpm, 4^o^C for 15 minutes. Amounts of endogenous PDGF-BB in the media and cell lysates were evaluated using a human PDGF-BB DuoSet Elisa kit (R and D Systems, USA) according to the manufacturer’s instructions. The optical density (OD) was immediately measured using a microplate reader set to at 450 nm, with a background correction wavelength of 540 nm. Normalized OD is defined as the ratio between the OD of tested samples and the OD of corresponding blanks. 

*Treatment of PDGF-BB and PDGF-BB aptamer*

To treat the PDGF-BB and PDGF-BB aptamer, CCD841 CoN, SW480 and Caco-2 cells were seeded into 96-well plates at a density of 5 x 10^3^ cells per well in complete media overnight. Then, media were removed, and the PDGF-BB and PDGF-BB aptamer were added at designated concentrations dependent on the experiments. To test the effect of PDGF-BB aptamer on cell proliferation, cells were incubated with 2.5, 12.5, and 25 nM PDGF-BB aptamer alone in DMEM with 5% FBS for 72 h. This concentration was equivalent to the one used in testing aptamer specificity. To determine the optimal concentration of exogenous PDGF-BB protein and time that can induce CRC cell proliferation, cells were incubated with 0, 10, 20, 30, and 40 ng/ml PDGF-BB in FBS-free media for 48 and 72 h. Finally, to investigate the direct effect of the aptamer on PDGF-BB-induced cell proliferation, cells were treated for 72 h with PDGF-BB that had been pre-incubated with aptamer at various molar ratios for 30 min before treatment. The ratio of PDGF-BB: aptamer varied from 1:0, 1:1, 1:5 to 1:10 in FBS-free media. The harvested cells were measured for cell viability using cell proliferation assays. 

*Cell proliferation assay*

Cell proliferation assays were performed by using CellTiter 96 Aqueous One Solution cell proliferation assay (Promega, USA). MTS solution was added to each well and incubated for 1 h in accordance with the supplier’s protocol. The absorbance of the solution was determined at 490 nm using a microplate reader (Bio Tek, USA).

*Protein extraction and Western blot*

Cells were treated with PDGF-BB with or without its aptamer. After cell harvesting, the pellets were washed three times with cold PBS, lysed with RIPA buffer (Ameresco, USA) containing protease inhibitors (Ameresco, USA), and sonicated using a sonicator. Protein concentrations were measured using a Pierce BCA protein assay kit (Thermo Scientific, USA). The cell lysates were subjected to polyacrylamide gel electrophoresis (PAGE, resolving gel 12% and stacking gel 4%). Then, the partitioned molecules were transferred to PVDF membrane by electrical blotting. The membranes were incubated with Odyssey blocking buffer for 1 h at room temperature, and then incubated with appropriate primary antibodies at 4^o^C overnight. After that, the membranes were incubated with accordant secondary antibodies at room temperature for 1 h. Band visualization and quantitation were carried out using a LI-COR Odyssey Imager (Li-COR, USA).

*Statistical analysis*

All data were represented as the mean ± standard deviation (SD) of at least three independent experiments done in triplicate. Comparisons among study groups were performed with Student’s t-test. Statistical significance was defined as *P < 0.05, **P < 0.01.

## Results

*CRC cells endogenously produce and secrete PDGF-BB*

**Figure 1:**
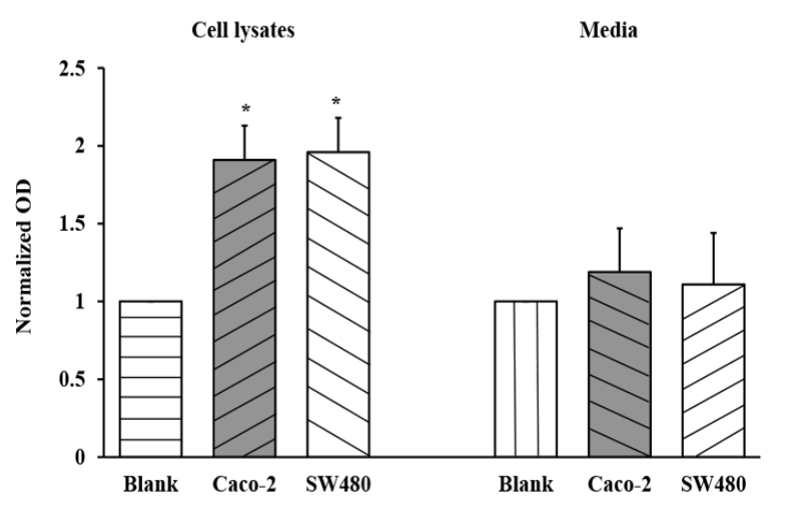
Verification of the PDGF-BB Endogenously Produced by Caco-2 and SW480 Cells in Culture Media and Cell Lysates. Blanks for culture media and cell lysates are DMEM and lysis buffer, respectively. The values are presented as mean ± SD, n = 3, *P < 0.05

To study the effects of the PDGF-BB aptamer on inhibition of CRC cell proliferation, Caco-2 and SW480 cells were selected to test with the PDGF-BB aptamer since these two cell lines have been confirmed to express the PDGF-BB receptors. Recent studies demonstrated that PDGFRß- mRNA and protein were expressed in both Caco-2 and SW480 cells. However, PDGFRα- mRNA and protein were found only in Caco-2 cells (Wehler et al., 2008; Kaulfuß et al., 2013). First of all, the endogenous production and secretion of PDGF-BB protein in these two cell lines were investigated. Caco-2 and SW480 cells were cultured in complete media for 72 h before the media and cell lysates were collected, and PDGF-BB levels were further analyzed using ELISA assay. The results showed that PDGF-BB could be significantly detected in cell lysates (Figure 1). From a calibration curve, the amount of PDGF-BB found in Caco-2 and SW480 were equivalent to 46.8, and 46.5 pg/ml, respectively. This experiment indicated that both Caco-2 and SW480 cells endogenously expressed PDGF-BB that bound to their receptors, causing the stimulation of cell growth in the autocrine pattern. 

*PDGF-BB aptamer decreases CRC cell proliferation*

**Figure 2:**
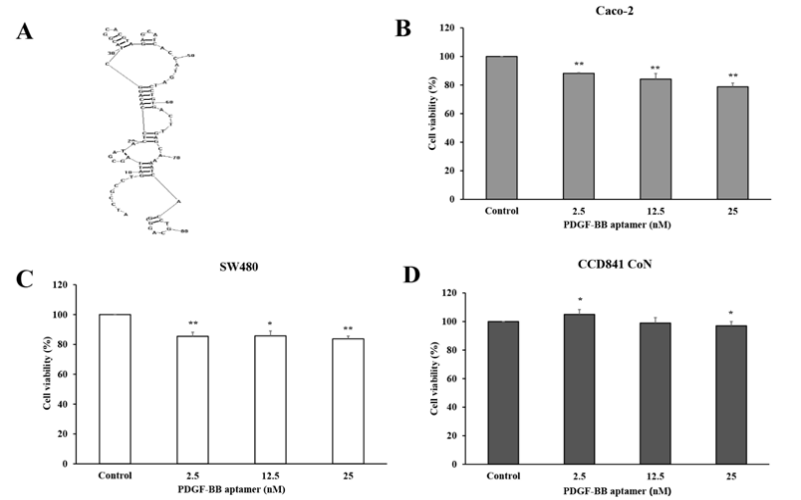
The PDGF-BB Aptamer Significantly Inhibits Caco-2 and SW480 but not CCD841 CoN Cell Proliferation. (A) The secondary structure of PDGF-BB aptamer. Caco-2 (B), SW480 (C) and CCD841 CoN (D) cells were treated with PDGF-BB aptamers (2.5, 12.5, and 25 nM) for 72 h, and cell viability was measured using MTS assay. The values are presented as mean ± SD compared with the control, n = 3, *P < 0.05 and **P < 0.01

From the literature, the PDGF-BB aptamer with 86 oligonucleotides in length was successfully developed (Green et al., 1996). The secondary structure of the aptamer was generated by RNAStructure version 6.0 (http://rna.urmc.rochester.edu/software.html) (Reuter and Mathews, 2010) (Figure 2A). Recently, the binding interaction between the aptamer and its target has been experimentally and computationally confirmed (Vu et al., 2017; Vu et al., 2018). To investigate the direct effect of PDGF-BB aptamer on CRC cell proliferation, Caco-2 and SW480 cells were treated with the aptamer at concentrations of 2.5, 12.5, and 25 nM in DMEM with 5% FBS for 72 h before assessing cell viability using MTS assay. As a result, the aptamer with the concentration of 25 nM highly inhibited both Caco-2 and SW480 cell proliferation (21.2% and 16.3% less proliferation as compared with the untreated cells, respectively) (Figures 2B-2C). Caco-2 cells demonstrated growth inhibition in a dose-dependent manner, yet SW480 did not show any significant difference among three concentrations. The results indicated that the PDGF-BB aptamer could reduce CRC cell proliferation by preventing the endogenous PDGF-BB function.

*PDGF-BB aptamer takes no effect on normal colorectal cell growth*

To study whether the PDGF-BB aptamer has an effect on normal colorectal cell growth, CCD841 CoN cells were treated with the aptamer at concentrations of 2.5, 12.5, and 25 nM in FBS-free media for 72 h. Although the cell proliferation assay showed that the PDGF-BB aptamer at 2.5 nM slightly induced CCD841 CoN cell proliferation, 12.5 nM and 25 nM aptamer very slightly reduced their proliferation compared with the control group (1% and 3%, respectively) (Figure 2D). The result suggested that the PDGF-BB aptamer had no cytostatic effect on normal colorectal cell growth, and vice versa, it was non-toxic to the normal cells.

*PDGF-BB promotes CRC cell proliferation*

**Figure 3:**
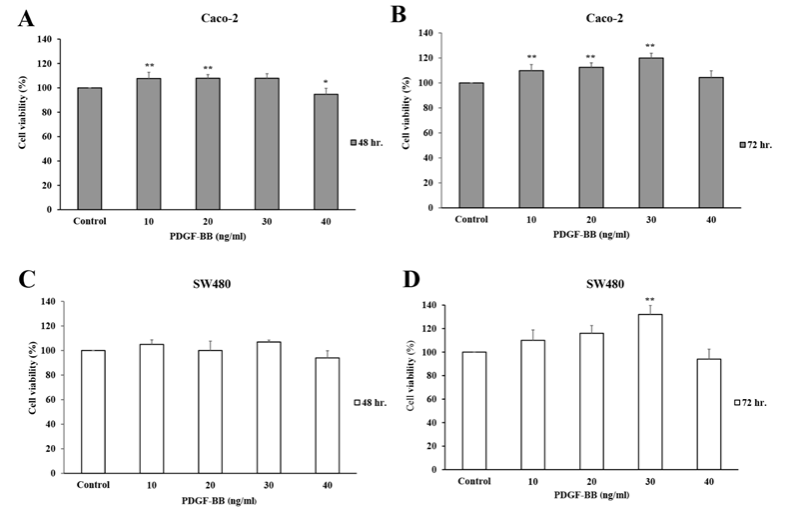
PDGF-BB at 30 ng/ml Significantly Stimulated Caco-2 and SW480 Cell Proliferation at 72 h. Caco-2 and SW480 cells were stimulated by PDGF-BB proteins at variable concentrations (10, 20, 30 and 40 ng/ml) for 48 h (A and C) and 72 h (B and D). The cell viability was measured using MTS assay. The values are presented as mean ± SD compared with the control, n = 3, *P < 0.05 and **P < 0.01

The inhibition of CRC cell proliferation, by silencing the endogenous PDGF-BB, still raised the question if the aptamer directly bound to the PDGF-BB and prevented cell proliferation. To find the answer, treatments of PDGF-BB with or without its aptamer into CRC cell lines were performed. However, the optimal concentration of PDGF-BB effectively stimulating CRC cell growth had to be explored first. Caco-2 and SW480 cells were treated with exogenous PDGF-BB at concentrations of 0, 10, 20, 30, and 40 ng/ml in FBS-free media for 48 and 72 h. The results showed that the PDGF-BB induced Caco-2 cell proliferation in a dose-dependent manner at 72 h (Figure 3B), but not at 48 h (Figure 3A). It most efficiently promoted cell proliferation, approximately 120% cell viability, at 30 ng/ml. In SW480 cells, the similar pattern of dose-dependent growth stimulation was observed at 72 h, and the optimal concentration was 30 ng/ml of PDGF-BB (Figure 3D). However, the cell viability of treated groups was not significantly different compared to the control at 48 h (Figure 3C). A minor decrease in cell growth was observed at 40 ng/ml for both 48 and 72 h in both cell lines. This might be explained by negative feedback of endogenous PDGF-BB production. From these results, the proliferation of Caco-2 and SW480 cells was effectively induced with 30 ng/ml PDGF-BB at 72 h.

*Specific targeting of PDGF-BB with its aptamer inhibits CRC cell proliferation*

**Figure 4:**
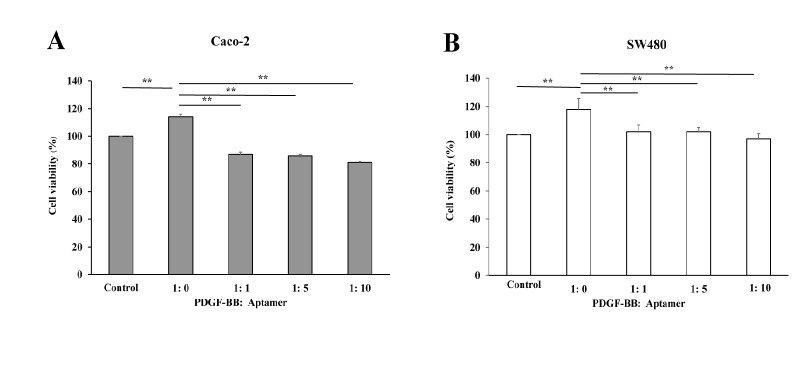
PDGF-BB Aptamer Effectively Abolished PDGF-BB-Induced Caco-2 and SW480 Cell Growth. Caco-2 (A) and SW480 (B) cells were treated with the PDGF-BB proteins previously incubated with the corresponding aptamers at the various molar ratios (PDGF-BB: Aptamer; 1:0, 1:1, 1:5 and 1:10) for 72 h. The cell viability was measured using MTS assay. The values are presented as mean ± SD, n = 3, **P < 0.01

According to the previous experiment, PDGF-BB at 30 ng/ml, approximately 2.5 nM, demonstrated the most effective induction of cell proliferation. To confirm whether the reduction in CRC cell growth was governed by the direct silencing of PDGF-BB with its aptamer, the various molar ratios between PDGF-BB and its aptamer (1:0, 1:1, 1:5 and 1:10) were treated to the cells. As shown in Figure 4A and 4B, pre-incubation of PDGF-BB with its aptamer significantly decreased Caco-2 and SW480 cell proliferation even at the least molar ratio, 1:1. The increase in molar ratio did not significantly multiply the inhibitory effect of aptamer. The results suggested that the PDGF-BB aptamer directly interacted with PDGF-BB, and subsequently prevented the growth factor from activating their receptors.

*Downstream PDGF-BB signaling pathway is suppressed by PDGF-BB aptamer*

**Figure 5:**
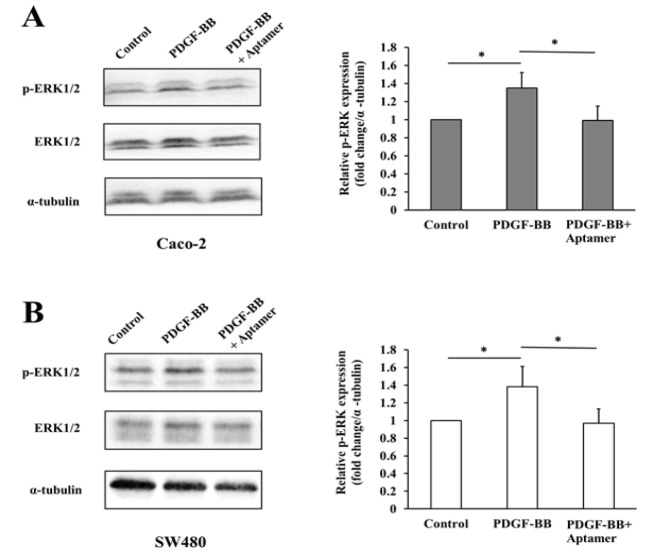
Blocking PDGF-BB with the Aptamer Decreases the Downstream Ras/Raf/MEK/ERK Signaling Pathway. Caco-2 (A) and SW480 (B) cells were treated with the PDGF-BB alone or PDGF-BB plus PDGF-BB aptamer, and the untreated cell served as control. The protein levels of phosphorylated ERK1/2 (p-ERK1/2), total ERK1/2, and α-tubulin were analyzed by Western blot. Bar graphs represent quantitative analysis of band intensities normalized as to α-tubulin. The values are presented as mean ± SD, n= 3, *P < 0.05

To elucidate the molecular mechanism underlying the inhibitory effect of PDGF-BB aptamer on cell proliferation, the changes in phosphorylation of ERK1/2 as a marker of Ras/Raf/MEK/ERK pathway and Akt as a key player of PI3K/PTEN/Akt/mTOR pathway were determined at various conditions by Western blot analysis. Caco-2 and SW480 cells were incubated with PDGF-BB only or the mixture of PDGF-BB and the aptamer for 24 and 48 h, respectively. As a result, the total protein levels of ERK1/2, Akt and α-tubulin in both cells were not significantly changed at any conditions. The phosphorylation of ERK1/2 was significantly increased after treatment of PDGF-BB compared to the untreated cells (Control); on the other hand, the elevated level of phosphorylated ERK1/2 was significantly suppressed in cells co-treated with the aptamer (Figures 5A-5B). However, the phosphorylation of Akt was undetectable in both cell lines (Supplementary Figure 1). This finding suggested that the specific blockade of the PDGF-BB with the concordant aptamer decreased CRC cell proliferation by diminishing downstream ERK1/2 signaling pathway.

## Discussion

PDGF-BB, a homodimer of PDGF-B, was detected at high levels in CRC (Nakamura et al., 2008). It plays at least three roles in tumorigenesis, including: (i) autocrine stimulation of cancer cells; (ii) induction of angiogenesis; (iii) regulation of tumor interstitial pressure (Marius Raica, 2010). Therefore, PDGF-BB is supposed to be a target for therapeutic intervention. Inhibitors of PDGF and/or PDGFR and some downstream targets have been developed and are currently in clinical trials. 

Aptamers are small single-stranded nucleic acids that fold into a well-defined three-dimensional structure. They show a high affinity and specificity for their target molecules and inhibit their biological functions (Mayer, 2009). They have no effect on immune response and complement stimulation (Bouchard et al., 2010; Lee et al., 2015). Moreover, chronic toxicity studies of aptamers at 5, 50, and 500 mg/kg/day in rats, as well as 0.75 and 7.5 mg/kg/day in woodchucks showed non-toxicity in terms of clinical chemistry and hematology (Richardson et al., 1999). 

In the present study, the aptamer specifically binding with the PDGF-BB was used to interfere the interaction between the PDGF-BB and corresponding receptors; consequently, the signal transduction was blocked and cell proliferation was inhibited subsequently. First of all, the endogenous PDGF-BB in cell lysates and secreted form in culture media of Caco-2 and SW480 cells were evaluated to confirm the autocrine stimulation of cell growth. The PDGF-BB level in cell lysates was detectable, since the proteins were synthesized within the cells and subsequently released outside. Since the levels of endogenous PDGF-BB are small, almost all secreted PDGF-BB might interact with its corresponding receptors. Thus the rest of secreted PDGF-BB level in culture media was not detectable. Next, the PDGF-BB aptamer was treated to the Caco-2 and SW480 cells to silence the endogenous PDGF-BB. Proliferations of both cell lines were significantly decreased, indicating anti-proliferative effect of the aptamer on CRC cells. On the other hand, the aptamer had no effect on normal colon cell growth. This might be explained by the low expression of PDGF and PDGF receptor in several normal epithelial cells, including in colon (Alexander et al., 1995; Heldin, 2013). However, it was curious whether the reduction in CRC cell growth was caused by direct silencing of PDGF-BB or due to the side inhibition of other molecules. To clarify this question, the proliferation rate of cells treated with PDGF-BB alone was compared with one treated with PDGF-BB that had been incubated with aptamer before. Caco-2 and SW480 cells were treated with various doses of PDGF-BB to find an optimal concentration that effectively stimulated the cell growth. As a result, 30 ng/ml PDGF-BB, approximately 2.5 nM, best promoted the proliferation rate at 72 h. Cells were incubated with a number of molar ratios between PDGF-BB and its aptamer (1:0, 1:1, 1:5 and 1:10). The results showed that cell proliferation was decreased (approximately 30%) even at the lowest molar ratio (1:1) compared to the one with PDGF-BB alone (1:0). Given that the utilized aptamer could bind to its target and block its subsequent function, this finding is consistent with the previous study that has demonstrated the specific interaction between this aptamer and PDGF-BB *in vitro* and *silico* (Vu et al., 2017). 

Tumor growth and metastasis are considered to be a consequence of complex, dysregulated signal transduction pathways (Lei et al., 2014). PDGF isoforms bind two distinct class III receptor tyrosine kinases, PDGFRα and PDGFRß. This interaction leads to autophosphorylation of the receptors on tyrosine residues and then activates several signaling molecules, such as STAT3, Akt, and ERK1/2 pathways (Yu et al., 2003; Yan et al., 2017). Nilotinib (PDGFR tyrosine kinase inhibitor) combined with everolimus (mTOR inhibitor) decreases cell growth and increases apoptosis of colon cancer (Yuge et al., 2015). The Ras/Raf/MEK/ERK and PI3K/PTEN/Akt/mTOR pathways are majorly involved in several biological processes such as proliferation, survival, angiogenesis, and metastasis and invasion of CRC cells (Cohen et al., 2005; Fang and Richardson, 2005; Steelman et al., 2011). Therefore, the phosphorylation levels of ERK1/2 and Akt proteins were further investigated to clarify the mechanism underlying the inhibition of cell proliferation. The protein expression was evaluated by western blot at 24 and 48 h, earlier than cell proliferation assay at 72 h because the alteration of protein level was occurred at molecular level. The change in cell signaling might be observed first, then the cellular behavior, in this case cell proliferation, would be subsequently noticed (Mebratu and Tesfaigzi, 2009). As a result, PDGF-BB activated the phosphorylation of ERK1/2, but no stimulation of Akt signal transduction was found in both CRC cell lines. The given aptamer suppressed PDGF-BB-induced phosphorylation of ERK1/2. This result suggests that the PDGF-BB aptamer could function as an anti-proliferative agent via downregulating downstream ERK1/2 signaling pathway. ERK1/2 is a mitogen-activated protein kinase (MAPK) pathway that is of great interest and importance to be the target in CRC treatment. Several studies have shown that overexpression of the ERK1/2 signaling pathway led to the progression of colorectal cancer (Fang and Richardson, 2005; Inoue et al., 2011; Ni et al., 2015). For example, parathyroid hormone-related peptide (PTHrP) induced colon cancer cell migration via upregulating ERK1/2 signaling pathway (Calvo et al., 2017). Piperlongumine, sodium butyrate, and ursodeoxycholic acid promoted apoptosis in human colon cancer by suppressing the ERK1/2 signaling pathway (Im and Martinez, 2004; Randhawa et al., 2013; Xiao et al., 2014). Furthermore, the blockade of the ERK1/2 signaling pathway suppressed the growth of colon cancer as much as 80% *in vivo* (Sebolt-Leopold et al., 1999). 

Recently, the aptamer has gained significant attentions in various research areas, including diagnostic and therapeutic applications. A previous study revealed that PDGF-BB aptamer plus bevacizumab inhibited proliferation of ovarian cancer more efficiently than bevacizumab alone because PDGF-BB aptamer also decreased vascular maturation (Lu et al., 2010). Furthermore, E10030 (PDGF aptamer) combined with ranibizumab was more effective than ranibizumab alone in the treatment of age-related macular degeneration (AMD) (Ni et al., 2011; Sadiq et al., 2016). Our study adds more evidence that the PDGF-BB aptamers are appropriate to use as drugs with high target specificity and fewer side effects in cancer treatment, including CRC. They could effectively prevent the interaction between the PDGF-BB and its receptor, subsequently decrease Ras/Raf/MEK/ERK signaling transduction, and eventually lead to a slower rate of cancer cell proliferation. 
